# Influence of a Physiologically Formed Blood Clot on Pre-Osteoblastic Cells Grown on a BMP-7-Coated Nanoporous Titanium Surface

**DOI:** 10.3390/biomimetics8010123

**Published:** 2023-03-16

**Authors:** Leonardo Raphael Zuardi, Cleide Lúcia Araújo Silva, Eduardo Magalhães Rego, Giovana Vacilotto Carneiro, Silvia Spriano, Antonio Nanci, Paulo Tambasco de Oliveira

**Affiliations:** 1 Department of Basic and Oral Biology, School of Dentistry of Ribeirão Preto, University of São Paulo, Ribeirão Preto 14040-904, SP, Brazil; 2 Haematology Division, Ribeirão Preto Medical School, University of São Paulo, Ribeirão Preto 14051-060, SP, Brazil; 3 Department of Applied Science and Technology, Politecnico di Torino, 10129 Torino, Italy; 4 Faculté de médecine dentaire, Université de Montréal, Montreal, QC H3T 1J4, Canada

**Keywords:** BMP-7, osteoblast, titanium, nanotopography, blood clot

## Abstract

Titanium (Ti) nanotopography modulates the osteogenic response to exogenous bone morphogenetic protein 7 (BMP-7) in vitro, supporting enhanced alkaline phosphatase mRNA expression and activity, as well as higher osteopontin (OPN) mRNA and protein levels. As the biological effects of OPN protein are modulated by its proteolytic cleavage by serum proteases, this in vitro study evaluated the effects on osteogenic cells in the presence of a physiological blood clot previously formed on a BMP-7-coated nanostructured Ti surface obtained by chemical etching (Nano-Ti). Pre-osteoblastic MC3T3-E1 cells were cultured during 5 days on recombinant mouse (rm) BMP-7-coated Nano-Ti after it was implanted in adult female C57BI/6 mouse dorsal dermal tissue for 18 h. Nano-Ti without blood clot or with blood clot at time 0 were used as the controls. The presence of blood clots tended to inhibit the expression of key osteoblast markers, except for *Opn*, and rmBMP-7 functionalization resulted in a tendency towards relatively greater osteoblastic differentiation, which was corroborated by runt-related transcription factor 2 (RUNX2) amounts. Undetectable levels of OPN and phosphorylated suppressor of mothers against decapentaplegic (SMAD) 1/5/9 were noted in these groups, and the cleaved form of OPN was only detected in the blood clot immediately prior to cell plating. In conclusion, the strategy to mimic in vitro the initial interfacial in vivo events by forming a blood clot on a Ti nanoporous surface resulted in the inhibition of pre-osteoblastic differentiation, which was minimally reverted with an rmBMP-7 coating.

## 1. Introduction

The functionalization of biomaterial surfaces with bioactive molecules is considered a promising strategy when specific cellular and/or tissue biological effects are desired [1,2]. An evaluation of the effectiveness of these surface modifications through clinical tests follows previous in vitro analyses and in vivo studies in animal models [3,4]. In vitro studies on the biological characterization of functionalized biomaterials have been developed using two- and three-dimensional cell culture models, which, at least theoretically, explain the complex phenomena of cell interactions in the context of interfacial tissue formation in a controlled environment [3,5]. However, importantly, most of these in vitro models do not take into account the formation of a physiological blood clot, simultaneously and/or prior to the adhesion and spreading of different cell types on the biomaterial surface. The addition of the formation of a three-dimensional fibrin clot to the model and the presence of functional leukocytes, platelets, plasma proteins, and proteases reduce the distance between the in vitro biological characterization and the in vivo reality of animal models and clinical tests [6,7].

Bone morphogenetic protein 7 (BMP-7) has been used in functionalization studies of biomaterials, due to its recognized stimulatory effects on osteogenic activity under specific conditions and the permission for its use in humans by the Food and Drug Administration [8,9]. The strategies used to functionalize with BMP-7 involve modifications of the biomaterial surface chemistry and topography at a nanoscale, including those of metals [8,9,10,11]. In a previous initial study, we observed that the effects of providing 40 and 200 ng/mL of a recombinant mouse (rm) BMP-7 to osteoblastic cells grown on titanium (Ti) were modulated by the nanotopographic features of its surface, with an increased expression of the osteoblast markers osteopontin (OPN) and alkaline phosphatase (ALP), but without enhancement in the mineralized matrix formation [12]. Interestingly, the Ti nanotopography (Nano-Ti) per se (without rmBMP-7 in the culture medium) promoted osteogenic differentiation compared with an almost flat surface at a nanoscale [12]. Based on these results, we designed the present study aiming to evaluate the effects of rmBMP-7-functionalized Nano-Ti on in vitro osteoblastic differentiation when a physiological blood clot is formed prior to the cell culture analyses.

We tested whether the presence of a blood clot alters key aspects of the interaction of osteoblastic cells with the nanotopographic surface with or without rmBMP-7 coating. As reported in the literature, the majority of studies that evaluate the interactions of blood clots with biomaterials use the simple addition of blood on their surfaces [13,14,15,16,17]. The present study goes further by including the formation of a physiological blood clot using the surgical approach proposed elsewhere [18] and adapted to our conditions to ensure the implant placement in a mouse dorsal dermal tissue model. The implant Ti discs were chemically treated with a mixture of sulfuric acid (H_2_SO_4_) and hydrogen peroxide (H_2_O_2_) to create a highly hydrophilic, biomimetic TiO_2_ nanotopography [19] supportive of (i) functionalization with organic molecules by physisorption [20] and (ii) osteogenic differentiation [12,21]. The average roughness of Nano-Ti has been reported to be on the order of 9 nm, with nanopore depths ranging from 5 to 25 nm [22,23]. The implant Ti discs were structured to limit the displacement of the blood clot.

## 2. Materials and Methods

### 2.1. Preparation of a Nanotopographic Titanium Surface (Nano-Ti)

To obtain Nano-Ti, commercially pure grade 2 Ti discs (Realum, São Paulo, Brazil), modified to exhibit a hollowed surface (Figure S1), were cleaned by sonication and etched in a solution consisting of equal volumes of concentrated 30% H_2_O_2_ and H_2_SO_4_ (95–97%) for 4 h (10 mL/disc) at room temperature (RT) [12,20]. Then, they were rinsed in distilled water, air dried, and autoclaved.

### 2.2. Functionalization of rmBMP-7 on Nano-Ti

Autoclaved Nano-Ti discs were incubated overnight at 4 °C in a solution of 400 ng/mL of rmBMP-7 (BP-5666, R&D Systems, Minneapolis, MN, USA), 0.1% BSA, and 4 mM HCl, and then washed three times with PBS at 37 °C [24].

### 2.3. Physiological Blood Clot Formation on Nano-Ti

The experimental protocol was approved by the Ethics Committee for the Use of Animals of Ribeirão Preto Medical School (FMRP-USP, Ribeirão Preto, Brazil), registered under the number 173/2016 (Figure S2). Female mice of the C57Bl/6 strain, GFP-positive, aged between 8 and 12 weeks, were used. The surgical model proposed by this study was based on Monroe and Hoffman [18], with adaptations. The animals were anesthetized with 3% isoflurane in an induction chamber, reaching a deep anesthetic, shaved on the posterior dorsal region, followed by antisepsis with povidone iodine (PVPI). A 250 mm incision was then made in the animal’s skin in the sagittal plane region and the muscular fascia was ruptured to facilitate hemorrhage. Thus, two Nano-Ti discs (functionalized with or without rmBMP-7) were introduced on both sides in the subcutaneous regions of each animal with the side of the hollowed surface facing the hemorrhage. The incision was stapled using veterinary surgical stapling (Autoclip 9 mm, Clay Adams, Vernon Hill, IL, USA). During and 6 h after surgery, the animals received a 40 mg/kg dose of the analgesic tramadol hydrochloride (Agener União, São Bernardo do Campo, Brazil), intraperitoneally (IP). After 18 h of blood clot formation, the animals were anesthetized with the administration of 5 mg/kg of 2% lidocaine hydrochloride (Lidovet, Engenho Novo, Brazil) IP and were sacrificed by a 150 mg/kg overdose of anesthetic with 1 g of sodium thiopental (Cristália, Itapira, Brazil) IP in order to remove the blood-clot-coated Nano-Ti discs. The discs and animals were organized into their intended respective groups, as shown in Table 1.

### 2.4. Pre-Osteoblastic MC3T3-E1 Cell Culture

The mouse pre-osteoblastic MC3T3-E1 cells, subclone 14 (CRL-2594, ATCC, Manassas, VA, USA), were used for a culture period of 5 days. Briefly, the cells were grown in T-75 culture flasks (Corning Inc., Kennebunk, ME, USA) in a 16 mL expansion medium composed of α-MEM (Invitrogen, Carlsbad, CA, USA), supplemented with 1% penicillin–streptomycin (Sigma-Aldrich, Saint Louis, MO, USA) and 10% fetal bovine serum (Invitrogen) at 37 °C in a humidified atmosphere containing 5% CO_2_. After subconfluence, the cells were removed with 1 mM ethylenediaminetetraacetic acid EDTA (Gibco, Grand Island, NY, USA) and 0.25% trypsin (Gibco), plated at a density of 5 × 10^4^ cells/well (~350 cells/mm^2^) on Nano-Ti discs placed in 24-well polystyrene plates (Corning Inc.), in an α-MEM culture medium supplemented with 5 µg/mL ascorbic acid and 7 mM β-glycerophosphate (Sigma-Aldrich). At days 2 and 4 of culture, the culture medium was changed (1 mL/well), as previously reported [12].

### 2.5. Cell Morphology by Epifluorescence Microscopy

The samples (MC3T3-E1 cultures and the control blood clots) were fixed in 4% paraformaldehyde in a 0.1 M phosphate buffer (PB; pH 7.2) at RT and treated with Triton X-100 at 0.5% in PB to permeabilization, before being labeled with 1:200 Alexa Fluor 594-conjugated phalloidin (A12381, Invitrogen) and 300 nM 4′,6-diamidino-2-phenylindole, dihydrochloride (DAPI, Molecular Probes, Eugene, OR, USA) [21]. The discs were mounted on glass slides (Thermo Fisher Scientific, Waltham, MA, USA) using a Vectashield anti-fade fluorescence mounting medium (Vector Laboratories, Burlingame, CA, USA). The images of the discs were acquired by an Axio Imager 2 fluorescence microscope (Carl Zeiss, Jena, Germany) using AxioVision 4.8.2 program, and processed in Adobe Photoshop CS6 (Adobe Systems, San Jose, CA, USA), as previously described [12].

### 2.6. Cell Morphology by Scanning Electron Microscopy (SEM)

The samples (MC3T3-E1 cultures and the control blood clots) were fixed in 2% glutaraldehyde (Electron Microscopy Sciences, Hatfield, UK) in a cacodylate buffer (0.9 mM Ca^2+^ and 0.5 mM Mg^2+^) for 2 h at RT, followed by two washes with 1% cacodylate buffer (EM Sciences) for 2 h at RT. Samples were then washed in Milli-Q water and incubated in saturated a thiocarbohydrazide solution (Electron Microscopy Sciences) for 10 min at RT, followed by five washes in Milli-Q water and incubation in 1% OsO_4_ (in Milli-Q water). The biological material was dehydrated in several baths with increasing concentrations of ethanol (30, 50, 70, 90, and 100%) and dried to the critical point with CO_2_ (Bal-Tec CPD 030 Critical Point Dryer, Balzers, Liechtenstein). Then, the samples were fixed with silver glue (EM Science) on a metallic support and covered with gold (Bal-Tec CPD 050 Sputter Coater). The samples were analyzed using a JEOL JSM-6610LV scanning electron microscope (JEOL, Tokyo, Japan) at the Multiuser Laboratory of Electron Microscopy (LMME- FMRP-USP) using 20 and 25 kV.

### 2.7. Quantitative Real-Time Polymerase Chain Reaction (Real-Time PCR)

Briefly, the total RNA was removed using TRIzol LS (Invitrogen) and purified using the SV Total RNA Isolation System kit (Promega, Madison, WI, USA). Then, it was quantified in the GeneQuant 1300 device (GE Healthcare, Cardiff, UK) and assessed for its integrity using the 2100 Bioanalyser (Agilent, Santa Clara, CA, USA) (Figures S3 and S4). cDNA was synthesized from 1 µg of total RNA by reverse transcription using the High-Capacity cDNA Reverse Transcription kit (Applied Biosystems, Foster City, CA, USA), following the manufacturer’s instructions. TaqMan probes (Applied Biosystems) (Table 2) were used to access the osteoblastic gene expression via the CFX96 device (Bio-Rad, Hercules, CA, USA), as previously detailed [12]. PCR reactions were performed in one biological replicate, resulting from the pooling of 16 independent wells/experimental replicates for each group, and with three technical replicates, at two different moments. The results were analyzed based on the value of the cycle threshold (Ct), and the normalization and relative quantification of the gene expression were performed by the 2^−ΔΔCT^ method [25]. The results were normalized by constitutive gene *Gapdh*, assigning 1 to the MC3T3-E1 group. No statistical testing was applied due to pooling [12].

### 2.8. Western Blotting (WB)

Briefly, the samples were processed in an ultrasonic bath (Misonix, Farmingdale, NY, USA) using a RIPA buffer. The total protein was quantified using the micro Lowry assay using the DC™ kit protein assay (Bio-Rad). Then, proteins were separated by SDS polyacrylamide gel electrophoresis (10%) and transferred to polyvinylidene difluoride (PVDF) membranes (Thermo Fisher Scientific). The membranes underwent the following incubations at RT: (1) 5% skimmed milk or BSA solution in TBS-T, according to the manufacturer’s recommendations; (2) primary monoclonal antibody anti-runt-related transcription factor 2 (RUNX2), 1:500 (#12556, Cell Signaling Technology Inc., Danvers, MA, USA); (3) primary anti-OPN monoclonal antibody MPIIIB10-1, 1:1000 (Hybridoma Bank, Iowa City, IA, USA); (4) primary polyclonal anti-phosphorylated suppressor of mothers against decapentaplegic (SMAD) 1/5/9 antibody, 1:1000 (#13820, Cell Signaling Technology Inc.); (5) primary polyclonal anti-GAPDH antibody, 1:1500 (sc-25778, Santa Cruz Biotechnology, Dallas, TX, USA). The membranes were analyzed in the G-Box (Syngene, Frederik, MD, USA). The number of band pixels was measured using GeneSys 1.6.9 (Syngene) and GeneTools 4.3.8 (Syngene), as previously detailed [12]. Western blotting was performed in one biological replicate, based on the pooling of 20 independent wells/experimental replicates for each group, and with one technical replicate. The results were normalized by constitutive protein GAPDH, assigning 1 to the MC3T3-E1 group. No statistical testing was applied due to the pooling [12].

## 3. Results

### 3.1. Epifluorescence and SEM Imaging

**Figure 1:**
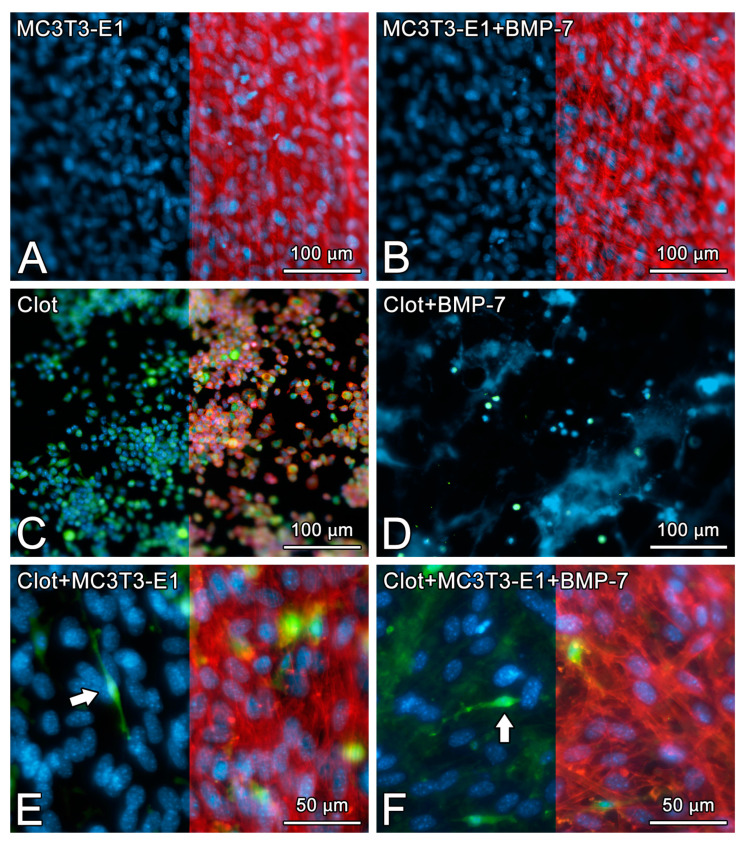
The epifluorescence of MC3T3-E1 cells on day 5 of culture (**A**,**B**), blood clot alone on time 0 (**C**,**D**), or the combination of both on day 5 of culture (**E**,**F**) on pristine Nano-Ti (**A**,**C**,**E**) or Nano-Ti functionalized with rmBMP-7 (**B**,**D**,**F**). Red fluorescence (Alexa Fluor 594 phalloidin) indicates the actin cytoskeleton (**A**–**C**,**E**,**F**), green fluorescence indicates the GFP-positive blood clot leukocytes (**C**–**F**), and blue fluorescence indicates the cell nuclei (DAPI nuclear stain) (**A**–**F**) and the fibrin network (**D**). Red fluorescence is deleted in the left halves of (**A**–**C**,**E**,**F**) using Adobe Photoshop CS6 for a better visualization of the cell nuclei and GFP-positive cells (white arrows in (**E**,**F**)) in the same microscopic field. Objectives: 20× (**A**–**D**); 40× (**E**,**F**).

**Figure 2:**
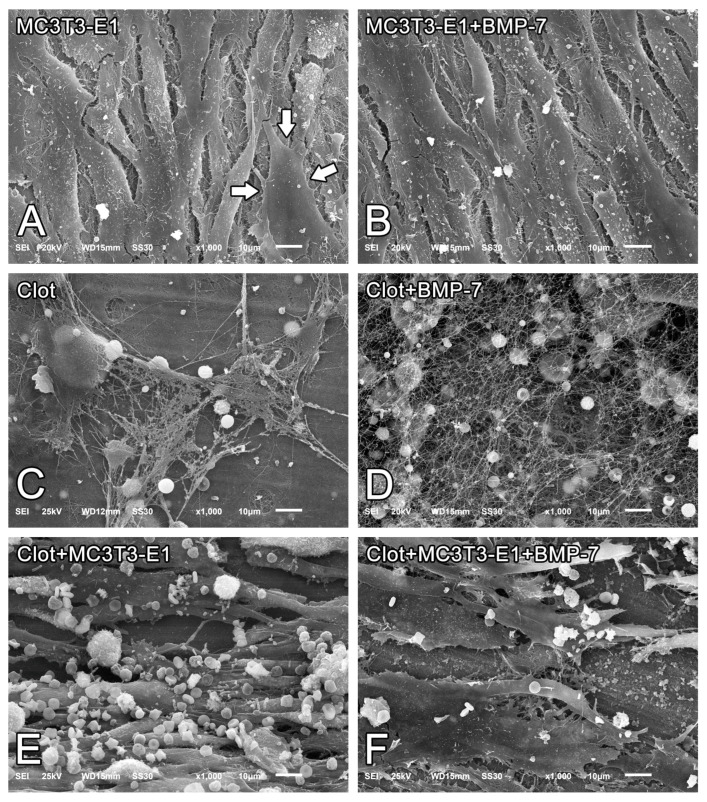
Scanning electron microscopy (SEM) imaging of MC3T3-E1 cells on day 5 of culture (**A**,**B**), blood clots alone on time 0 (**C**,**D**), or the combination of both on day 5 of culture (**E**,**F**) on pristine Nano-Ti (**A**,**C**,**E**) or Nano-Ti functionalized with rmBMP-7 (**B**,**D**,**F**). Original magnification: 1000× (**A**–**F**). MC3T3-E1 cells exhibit elongated shapes (white arrows in (**A**)) irrespective of the presence of a blood clot.

On day 5 of culture, MC3T3-E1 cells that were grown directly on Nano-Ti surfaces with or without rmBMP-7 functionalization were confluent, showing the beginning of multilayer formation and occasional mitotic figures (Figure 1A,B), exhibiting elongated shapes with occasional cytoplasmic projections (Figure 2A, arrows, and Figure 2B). Blood clot formation on Nano-Ti showed a complex structure, with an extensive three-dimensional fibrin network with which red blood cells, leukocytes, and platelets interacted (Figure 1C,D and Figure 2C,D). These elements persisted with MC3T3-E1 culturing, but in smaller amounts compared with pre-osteoblastic cell layers. MC3T3-E1 cells maintained their elongated shapes when intermingled with the blood clot structure, showing larger cytoplasmic and nuclear dimensions compared with the GFP-positive leukocytes. Leukocytes and erythrocytes were clearly visible in the upper plane of the cultures (Figure 1E,F and Figure 2E,F). Leukocytes exhibited either typical features of cell spreading, with cytoplasmic extensions, or a spherical morphology. No morphological changes that could be attributed to functionalization with rmBMP-7 were noticed in any of the groups (Figure 1 and Figure 2, compare B with A, D with C, and F with E).

### 3.2. Quantitative mRNA Expression by Real-Time PCR

**Figure 3:**
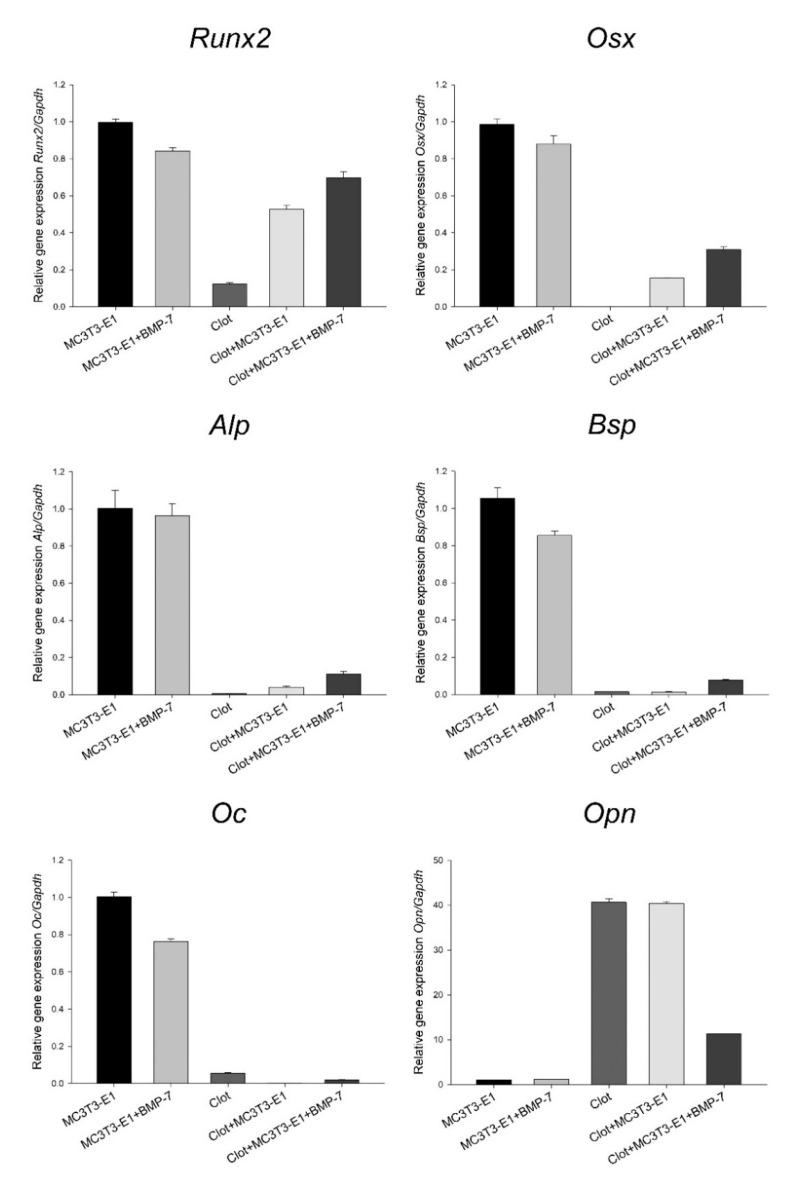
*Runx2*, *Osx*, *Alp*, *Bsp*, *Oc*, and *Opn* mRNA expression levels normalized to *Gapdh* in MC3T3-E1 cell cultures grown for 5 days and in blood clots on time 0 on Nano-Ti surfaces either functionalized with or without rmBMP-7. The bars represent one biological replicate (resulting from the pooling of 16 independent wells/experimental replicates for each group) run in three technical replicates (mean and SD). The mean value of the control MC3T3-E1 group is assigned a value of 1. Additional technical replicates obtained in a different moment using the same cDNA result in a similar expression pattern (Figure S5).

The results were presented as trends, as no statistical testing was applied due to the pooling of 16 independent experimental replicates for each group. The presence of blood clots altered the mRNA expression levels of key osteoblast markers in MC3T3-E1 cells grown on Nano-Ti, with a trend towards the inhibition of *Runx2*, osterix (*Osx*), *Alp*, bone sialoprotein (*Bsp*), and osteocalcin (*Oc*), except for *Opn*. Very low or minimal expression levels of these genes were detected in the clot group, while its *Opn* expression was high (Figure 3). Functionalization with rmBMP-7 resulted in a tendency towards a relatively small increase in the expression levels of these genes (about 0.3-fold for *Runx2*, 1.1-fold for *Osx*, 1.8-fold for *Alp*, and 4.8-fold for *Bsp*). The direct growth of MC3T3-E1 cells on Nano-Ti functionalized with rmBMP-7 supported lower *Runx2*, *Osx*, *Alp*, *Bsp*, and *Oc* levels compared with the ones on the pristine Nano-Ti (Figure 3).

### 3.3. Protein Detection by WB

**Figure 4:**
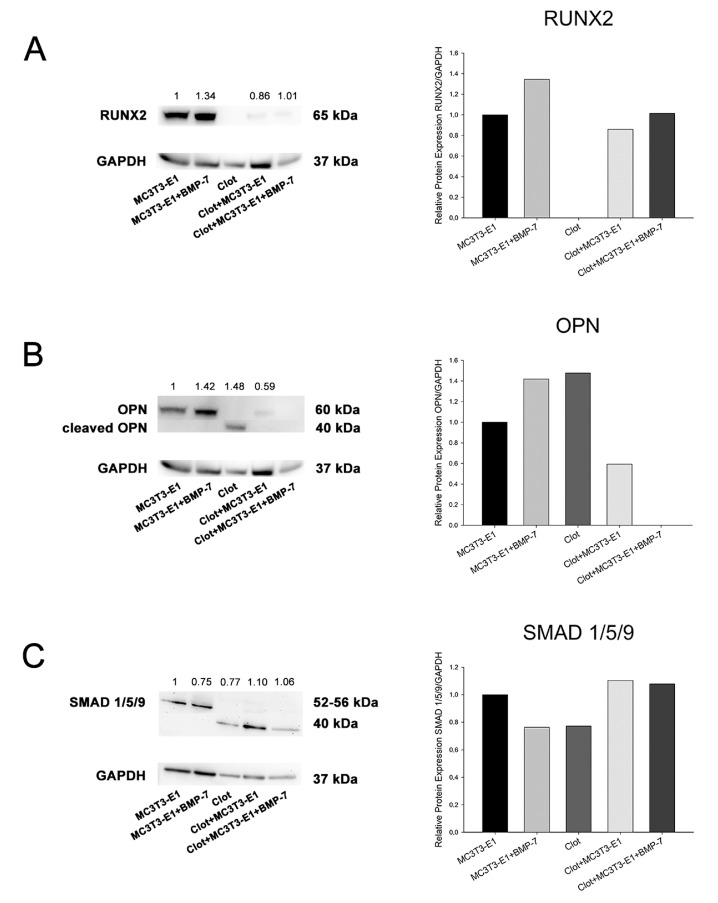
Detection and quantification of RUNX2 (**A**), OPN (**B**), and phosphorylated-SMAD 1/5/9 (**C**) by Western blotting (number of pixels of the protein bands normalized to GAPDH) in MC3T3-E1 cell cultures grown for 5 days and in blood clots on time 0 on Nano-Ti surfaces either functionalized with or without rmBMP-7. The bars represent one biological replicate (resulting from the pooling of 20 independent wells/experimental replicates for each group) and one technical replicate. The ratio of the control group (MC3T3-E1) is assigned a value of 1. Lower molecular weight bands in (**B**,**C**) are cropped to fit the figure.

The results represented one technical replicate from the pooling of 20 independent experimental replicates for each group, and thus were presented as trends. An increase in RUNX2 was observed in MC3T3-E1 cells on Nano-Ti functionalized with rmBMP-7, irrespective of the presence of blood clots. Prior to cell plating, the blood clots showed no detectable amounts of RUNX2 (Figure 4A). An increase in OPN was detected in cultures grown on Nano-Ti coated with rmBMP-7 in the absence of blood clots, while no detectable amounts were noted when the cells interacted with blood clots pre-formed on rmBMP-7-coated Nano-Ti (Figure 4B). A cleaved form of OPN was only detected in the blood clots at time 0, immediately prior to cell plating (Figure 4B). The amounts of phosphorylated-SMAD 1/5/9 were reduced by about 20% in cultures grown on Nano-Ti coated with rmBMP-7. For the blood clot groups—on time 0 and on day 5—antibody #13820 detected a nonspecific band with a lower molecular weight, which was in higher amounts with the presence of MC3T3-E1 cultures (Figure 4C).

## 4. Discussion

Our results indicate that the presence of a blood clot has an impact on some key aspects of the interaction of osteoblastic cells with a Ti surface nanotopography functionalized with or without rmBMP-7. There is a tendency towards inhibition of key osteoblast markers relating to mRNA expression, except that of *Opn*. As for the blood clot groups, the functionalization with rmBMP-7 promotes a tendency towards relatively greater osteoblastic differentiation, a finding that is corroborated by RUNX2 protein detection; as for the other proteins evaluated, undetectable levels of OPN and phosphorylated SMAD 1/5/9 are found in cultures exposed to rmBMP-7. The cleaved form of OPN is only detected in the ex vivo blood clot prior to MC3T3-E1 cell plating.

Functionalization with rmBMP-7 of Nano-Ti discs not implanted in the animals does not promote osteogenic differentiation of pre-osteoblastic MC3T3-E1 cells during the proliferative phase of cultures. In fact, two out of the six osteoblast markers evaluated—*Bsp* and *Oc*—exhibit a tendency in the order of 20% towards a reduced expression. Because of the 5-day culture time, it is not possible to predict the outcomes of the cultures in terms of osteogenic potential. The surface functionalization with rmBMP-7 likely causes a burst release of the exogenous growth factor, providing an extracellular BMP-7 concentration within the range that has been shown to be inhibitory for osteogenic differentiation [26,27,28]. However, this simple functionalization strategy must be considered with caution in light of other successful ones using either minimal or much higher concentrations of BMP-7 [8,9,10,11] On the other hand, it permits estimating the challenges that arise when one tries to mimic the interfacial in vivo reality for the design of in vitro studies on cell response to organic coatings on Ti. Our results highlight that it is feasible to evaluate the interactions of pre-osteoblastic cells with a Ti surface, and likely those with other solid biomaterials, and more closely mimic the in vivo reality by integrating a physiological blood clot prior to cell culture.

The strategy to create a hollowed surface on the Nano-Ti disc allows for the formation and stabilization of a blood clot in the mouse dorsal skin dermal tissue model. Epifluorescence and SEM imaging show that structurally, the clot consists of a three-dimensional fibrin network embedding variable amounts of platelets, leukocytes, and erythrocytes, distributed inhomogeneously throughout the surface. Although the combination of nanotopography and hydrophilicity (two surface characteristics of Nano-Ti) [20,21] might contribute to pronounced blood coagulation [29], it has been shown that implant surfaces structured at the micro-scale are more efficient at retaining the fibrin clot, an event that is crucial for bone repair to occur at the tissue–implant interface [30]. Therefore, considering the only occasional occurrence of microtopographic features on the Nano-Ti surface (not shown) and the inhomogeneity of the blood clot on it, it may be more appropriate to hierarchically structure the Ti surface at the micro- and nanoscale [31], or to even functionalize it with a molecule that could contribute to the formation of a more homogenous blood clot/fibrin clot in vivo. Either plasma-derived or recombinant fibrinogen might be a good candidate for that purpose.

Irrespective of the presence of rmBMP-7 coating, the observed inhibition of osteoblastic differentiation when MC3T3-E1 cells interact with the physiological blood clot likely reflects the functionality of blood cells and plasma proteins in this model, with synergistic pro-proliferative and/or anti-differentiation effects on pre-osteoblastic cells. More specifically, these effects could be attributed to the degradation rate of fibrin (the main matrix constituent of a blood clot), which impedes osteogenic differentiation under physiological concentrations of fibrinogen and thrombin, as discussed in [32]. In vitro, thrombin stimulates osteoblast cell proliferation, migration, and adhesion, and inhibits its differentiation, with effects that are mostly mediated by protease-activated receptor-1, as reviewed in [33]. Moreover, platelets, a major cellular component in blood clots, both trigger thrombin generation [34] and secrete a series of soluble factors that have been shown to inhibit osteoblastic cell differentiation. These include transforming growth factor beta (TGF-b), platelet-derived growth factor (PDGF), and thrombospondin [35,36,37,38]. Interestingly, at day 5 of culture, occasional GFP-positive leukocytes derived from blood clots are still observed and intermingled with MC3T3-E1 cells. This observation reveals a somewhat similar occurrence of the complex interplay between the immune system and bone cells that regulates osteoblastic cells at the onset of bone repair by transiently promoting their proliferation while limiting their differentiation, as reviewed in [39,40].

Contrarily to the other osteoblast markers studied, *Opn* expression tends to be upregulated in MC3T3-E1 cells grown on blood clots compared with those grown directly on Nano-Ti. Although it is well known that the overexpression of *Opn* inhibits osteoblast differentiation [41], this finding is of particular interest in the context of our biomimetic model, as the biological functions of OPN protein are largely determined by its proteolytic cleavage by the plasma proteases thrombin and plasmin, among others [42,43,44]. Interestingly, the cleaved form of OPN is only detected in the blood clot at time zero, prior to MC3T3-E1 cell plating, and is thus associated with the cytoplasmic and extracellular accumulation of the protein (derived either from plasma or cellular secretion) that occurs during blood clot formation in the mouse. For MC3T3-E1 cultures that interact with the blood clot during the 5-day period, full-length OPN is either detected in minimal amounts or is not detectable (for the rmBMP-7-coated Nano-Ti). One possible explanation for the discrepancy between mRNA and protein levels here is that OPN accumulation during the initial hours and days of cultures is limited due to protease activities. In addition, close to day 5 of culture, the absence of the cleaved form of OPN is indicative of protease inactivation [45,46].

Despite the benefits of simulating in vitro the initial tissue response to rmBMP-7-functionalized Nano-Ti, particularly including a physiological blood clot on its surface, the experimental model presented here exhibits some methodological challenges that remain to be addressed. Firstly, our strategy to use phosphorylated SMAD 1/5/9 as a marker of BMP/Smad signaling, as discussed in [47], surprisingly results in the detection of a nonspecific band with a lower molecular weight only on the blots related to the blood clot groups. At this point, one cannot rule out the possibility that it represents another SMAD [48] that cross-reacts with antibody #13820 and/or reveals the occurrence of the proteasomal degradation of SMAD 1/5/9 [49]. Secondly, the response of the cell line to the control surfaces is not totally comparable to that of the surfaces previously covered by blood clots. Coating with rmBMP-7 might be subjected to changes in its structure and composition, resulting from desorption events [50] and plasma protease activities [51], among others, while the discs are implanted in the animal. Finally, in order to further improve this biomimetic model, the relative proportions of cells of the osteoblast lineage and blood clot elements that occur in vivo at the bone–implant interface should be taken into consideration in order to establish a more realistic initial osteogenic cell population at cell plating.

## 5. Conclusions

The strategy to more closely simulate the early in vivo environment at the Ti interface using physiological blood clot formation reveals major changes at the morphological and molecular levels during the proliferative phase of pre-osteoblastic cell cultures on a nanostructured Ti surface functionalized with or without rmBMP-7. By growing MC3T3-E1 cells in the presence of a functional blood clot structure, osteoblastic differentiation tends to be inhibited, and this inhibition is minimally reverted with rmBMP-7 functionalization. These results highlight the importance of developing in vitro methods in studies of biomaterials that come closer to the in vivo reality. In addition, they point towards the challenges that arise in the attempt to functionalize Ti surfaces with BMP-7, or even other growth factors or organic molecules, in order to efficiently promote osteogenic differentiation.

## Figures and Tables

**Table 1:** Descriptive table of the number of animals and discs per group and per analysis. Western blotting (WB); real-time polymerase chain reaction (real-time PCR); morphology (epifluorescence and scanning electron microscopy).

Groups	Animals per Group	Animals per Analysis	Discs per Analysis
MC3T3-E1	-	-	WB (20) PCR (16) Morphology (4)
MC3T3-E1 + BMP-7	-	-	
Clot *	20	WB (10) PCR (8) Morphology (2)	
Clot + MC3T3-E1	20		
Clot + MC3T3-E1 + BMP-7	20		
TOTAL	60	60	200

* Additional 2 animals/4 BMP-7-coated Nano-Ti discs were used for morphological analyses of the Clot + BMP-7 group.

**Table 2:** TaqMan probes used in real-time PCR analysis.

Genes	Taqman Probes
*Runx2*	Mm00501584_m1
*Osx*	Mm04933803_m1
*Alp*	Mm00475834_m1
*Bsp*	Mm00492555_m1
*Oc*	Mm03413826_mH
*Opn*	Mm00436767_m1
*Gapdh*	Mm99999915_g1

## Data Availability

The data present in this study are available on request from the corresponding author under plausible justification.

## Supplementary Materials

The following supporting information can be downloaded at: https://www.mdpi.com/article/10.3390/biomimetics8010123/s1, Figure S1: Technical drawing of the modified titanium disc; Figure S2: Certificate of the local animal ethical committee; Figure S3: RNA integrity—Electrophoresis file run summary; Figure S4: RNA integrity—Electropherogram summary; Figure S5: Quantitative mRNA expression—Additional technical replicates.
