# Supplementary material for: Influence of a Physiologically Formed Blood Clot on Pre-Osteoblastic Cells Grown on a BMP-7-Coated Nanoporous Titanium Surface
Source: Biomimetics (Basel). 2023 Mar 16;8(1):123. doi: 10.3390/biomimetics8010123 (PMC10046195; doi:10.3390/biomimetics8010123)
Supplement: Supplementary file 1 [file biomimetics-08-00123-s001.zip › biomimetics-2235334-supplementary.pdf]

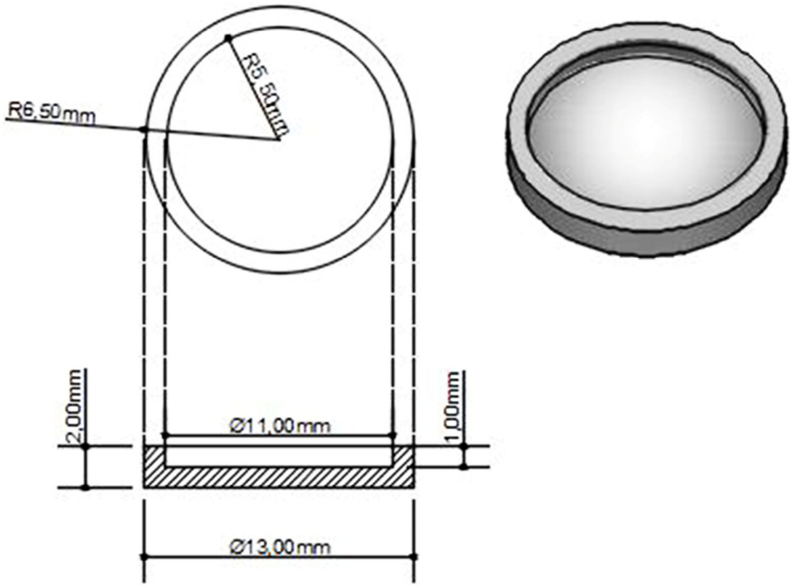

Figure S1. Technical drawing of the modified titanium disc.

Leonardo

UNIVERSIDADE DE SÃO PAULO
FACULDADE DE MEDICINA DE RIBEIRÃO PRETO

COMISSÃO DE ÉTICA NO USO DE ANIMAIS

CERTIFICADO

Certificamos que o Protocolo intitulado *"Efeitos do coágulo sanguíneo sobre a diferenciação de células osteoblásticas crescidas sobre nanotopografia de titânio funcionalizada com BMP-7"*, registrado com o número **173/2016**, sob a responsabilidade do **Prof. Dr. Eduardo Magalhães Rego,** envolvendo a produção, manutenção ou utilização de animais pertencentes ao *filo Chordata, subfilo Vertebrata* (exceto humanos) para fins de pesquisa científica, encontra-se de acordo com os preceitos da Lei nº 11.794 de 8 de outubro de 2008, do Decreto nº 6.899 de 15 de julho de 2009 e com as normas editadas pelo Conselho Nacional de Controle de Experimentação Animal (CONCEA), e foi **APROVADO** pela Comissão de Ética no Uso de Animais da Faculdade de Medicina de Ribeirão Preto da Universidade de São Paulo em reunião de 28 de novembro de 2016.

Este Protocolo prevê a utilização de 10 camundongos C57Bl/6 fêmeas pesando 22g, oriundos do Serviço de Biotério da Prefeitura do Campus de Ribeirão Preto da Universidade de São Paulo. Vigência da autorização: 28/11/2016 a 22/11/2020.

We certify that the Protocol *nº 173/2016*, entitled ***"Effects of blood clot formation on osteoblastic cell differentiation on a BMP-7- functionalized nanostructured titanium surface"***, is in accordance with the Ethical Principles in Animal Research adopted by the National Council for the Control of Animal Experimentation (CONCEA) and was approved by the Local Animal Ethical Committee from Ribeirão Preto Medical School of the University of São Paulo in *11/28/2016*. This protocol involves the production, aintenance or use of animals from *phylum Chordata, subphylum Vertebrata* (except humans) for research purposes, and includes the use of 10 female C57Bl/6 mice weighing 22g, from the Central Animal House of Ribeirão Preto Medical School, University of São Paulo. This certificate is valid until 11/22/2020.

Ribeirão Preto, 28 de novembro de 2016

Leonardo FORP

Prof. Dr. Fernando Silva Ramalho
Presidente da CEUA – FMRP – USP

Comissão de Ética no Uso de Animais da Faculdade de Medicina de Ribeirão Preto da Universidade de São Paulo
(16)3315-3301

UNIVERSIDADE DE SÃO PAULO
FACULDADE DE MEDICINA DE RIBEIRÃO PRETO
COMISSÃO DE ÉTICA NO USO DE ANIMAIS

CEUA FMRP-USP
Comissão de Ética no Uso de Animais
Replacement|Reduction|Refinement

FMRP 65 anos USP

DECLARAÇÃO

Declaramos que o Adendo ao Protocolo para Uso de Animais em Experimentação número **173/2016** sobre o projeto intitulado *"Efeitos do coágulo sanguíneo sobre a diferenciação de células osteoblásticas crescidas sobre nanotopografia de titânio funcionalizada com BMP-7"*, sob a responsabilidade do **Prof. Dr. Eduardo Magalhães Rego,** está de acordo com os Princípios Éticos em Experimentação Animal adotado pelo Conselho Nacional de Controle de Experimentação Animal (CONCEA) e foi **APROVADO** *ad referendum* na data de 28 de janeiro de 2019.

O presente adendo refere-se à solicitação de 72 camundongos C57Bl/6J fêmeas pesando 20g.

We declare that the Addendum to the Protocol *nº* 173/2016, entitled ***"Effects of blood clot formation on osteoblastic cell differentiation on a BMP-7- functionalized nanostructured titanium surface"***, is in accordance with the Ethical Principles in Animal Research adopted by the National Council for Control of Animal Experimentation (CONCEA) and was approved *ad referendum* in *01/28/2019* by the Local Animal Ethical Committee from the Ribeirão Preto Medical School of the University of São Paulo. This Addendum refers to the request of 72 female C57Bl / 6J mice weighing 20g.

Ribeirão Preto, 28 de janeiro de 2019

Prof. Dr. Fernando Silva Ramalho
Coordenador da CEUA – FMRP – USP

Faculdade de Medicina de Ribeirão Preto - USP - Av. Bandeirantes, 3900 - Ribeirão Preto - SP - Brasil -14049-900 - Tel.: (16) 3315-3301 / 3315.3275 - e-mail: ceua@fmrp.usp.br

Figure S2. Certificate of the local animal ethical committee.

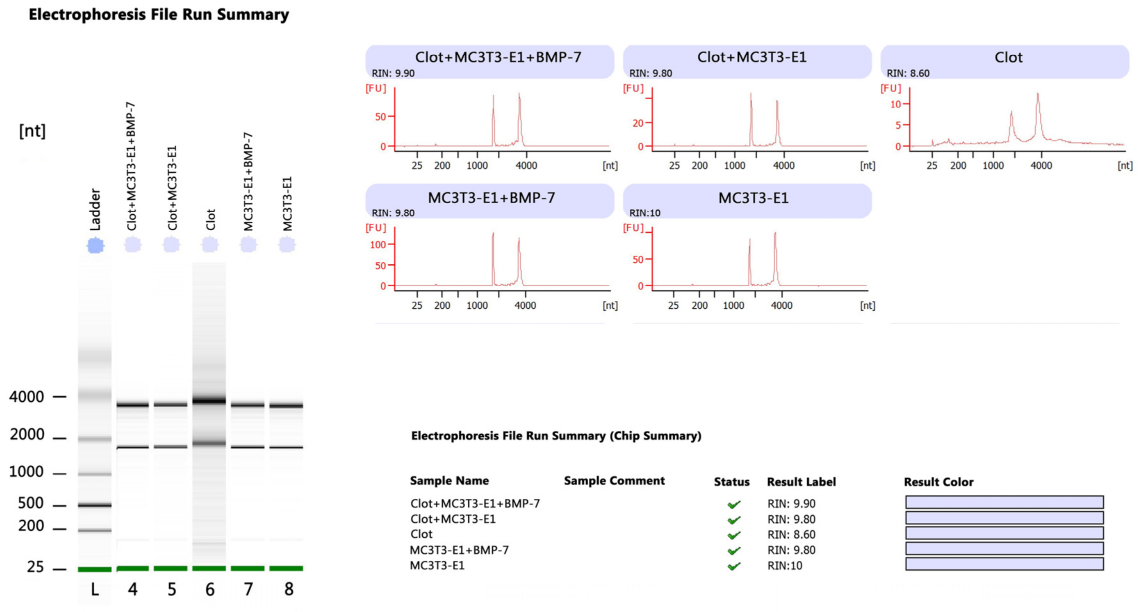

Figure S3. RNA integrity—Electrophoresis file run summary. Figure.

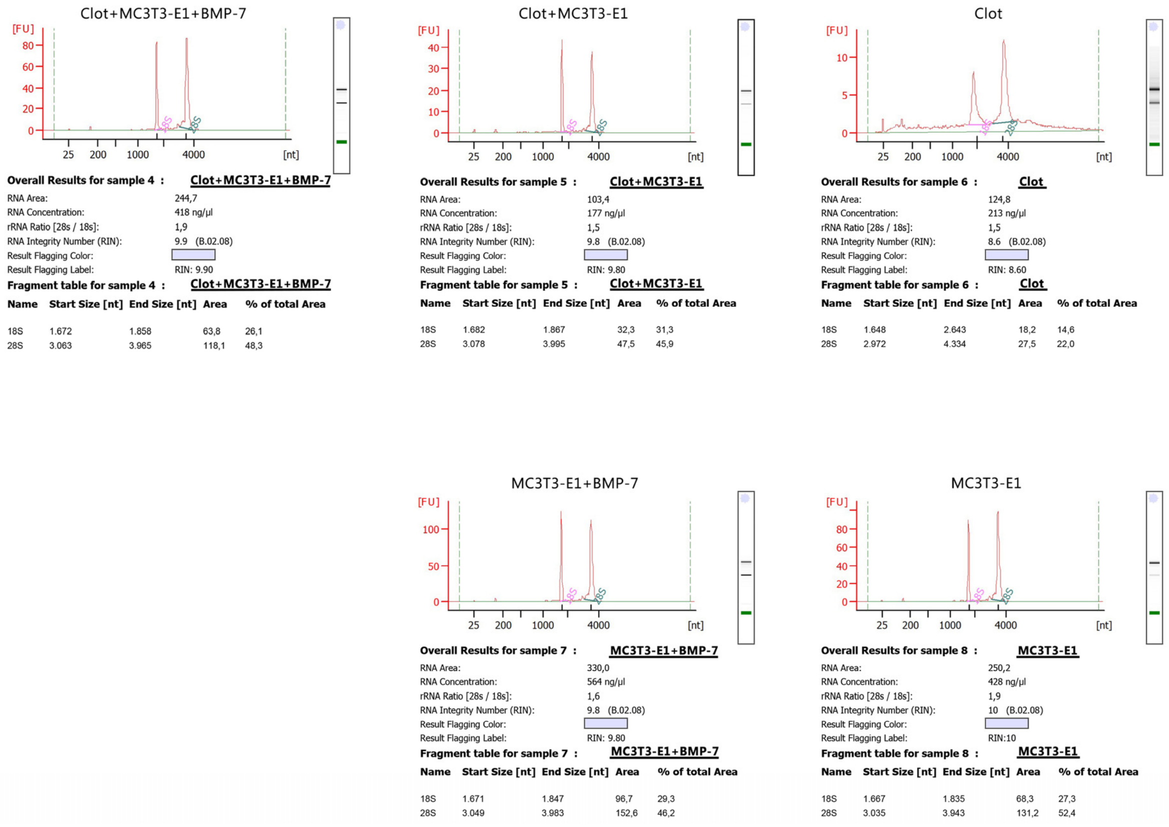

Figure S4. RNA integrity—Electropherogram summary.

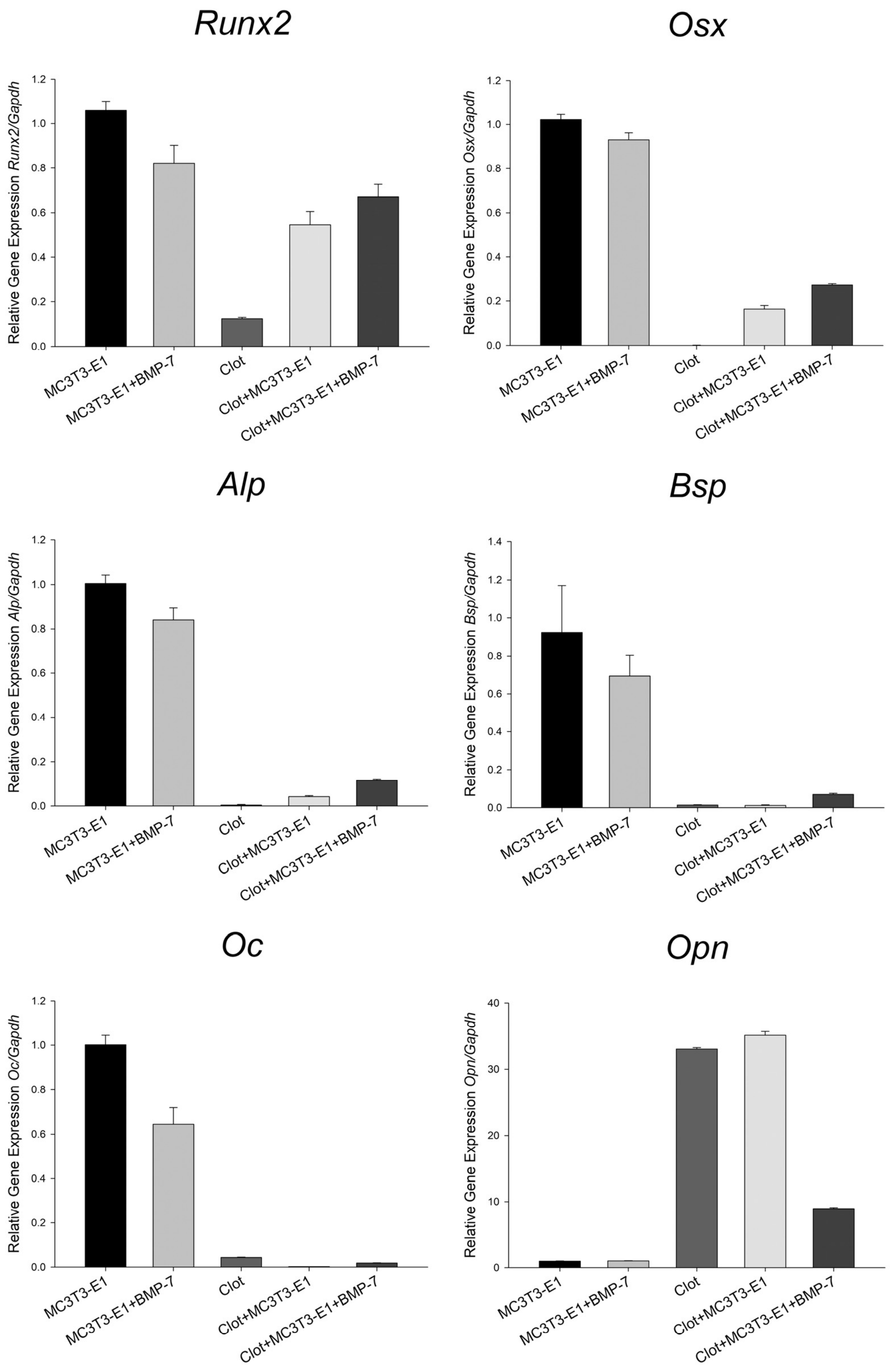

Figure S5. Quantitative mRNA expression—Additional technical replicates.
